# Rational Design of Crown Ether‐Based Fluorescent Sensors for Group II Cations: Insights From Vibrational Spectroscopy and Computation

**DOI:** 10.1002/cphc.202500770

**Published:** 2026-06-29

**Authors:** Bruno Martinez‐Haya, Jennifer C. Anene, Laura Finazzi, Jos Oomens, Simon Wheeler

**Affiliations:** ^1^ Department of Physical, Chemical and Natural Systems Universidad Pablo de Olavide Sevilla Spain; ^2^ Leicester School of Pharmacy De Montfort University Leicester UK; ^3^ Institute for Molecules and Materials FELIX Laboratory Radboud University Nijmegen The Netherlands

**Keywords:** density functional theory, fluorescence, group II, mass spectrometry, vibrational spectroscopy

## Abstract

Rational design of fluorescent sensors requires knowledge of the molecular structure of the sensor–analyte complex. We use infrared spectroscopy on isolated sensor‐cation complexes allied with density functional theory (DFT) calculations to reveal, for the first time, solution phase structures of crown ether‐based fluorescent sensors for group II cations. We show that similar fluorescent responses can arise from different ion binding modes thereby rationalizing the observed differences in UV absorption spectra in benchmark sensors. Finally, we are able to outline a structural explanation for the dramatic water sensitivity of the fluorescent response of these sensors to Ca^2+^.

## Graphical abstract

We use vibrational spectroscopy to determine the solution phase structures of complexes of two fluorescent sensors with metal ions. We use these to understand differences in the absorption spectra and to explain why even very low levels of water destroy the fluorescent response.

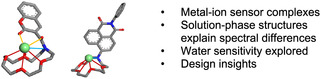

## 1 Introduction

The discovery of crown ether‐based fluorescent sensors for metal ions represents an ongoing area of work in supramolecular chemistry [1]. The two most commonly used mechanisms for such sensors to translate metal binding at the crown ether ionophore into a fluorescent signal are photo‐induced electron transfer (PET) and intramolecular charge transfer (ICT). In PET‐based sensors, electrons are transferred from the crown to the electronically excited state of an appended fluorophore thereby quenching fluorescence. Metal ion binding reduces this transfer meaning that fluorescence is enhanced. ICT‐based sensors typically employ an aza‐crown with the nitrogen conjugated to a fluorophore; metal ion binding alters this conjugation leading to changes in fluorescence.

The rational design of such sensors is a significant challenge, not least because of the lack of systematic structure–activity studies in this area, but also because previous work has produced results that are not always easy to harmonize. For example, the fluorescence of an N‐aryl aza‐15‐crown‐5 ICT sensor was enhanced ca. 200x on the addition of Ca^2+^ (ref. [2]), but the same ion‐binding unit bearing a different fluorophore gave a compound whose fluorescence was reduced by addition of the same ion [3]. Progress is likely to require a technique that elucidates the structure of metal‐sensor complexes with high resolution. We have previously used vibrational spectroscopy to study crown ether complexes with alkaline‐earth and other divalent metals [4], as well as with small molecular ions, including hydronium and ammonium ions [5, 6]. Such studies seek the characterization of the host‐guest configurations of lowest energy and a rationalization of cation recognition from first principles, based on the intrinsic intra‐ and intermolecular interactions and solvent effects [7, 8, 9, 10]. These investigations are framed within a solid body of work developed over decades by groups worldwide that have addressed the ionophoric behavior of crown ethers and their N‐substituted aza‐analogs from different fundamental perspectives and with different technological applications in view [11, 12, 13, 14].

**FIGURE 1:**
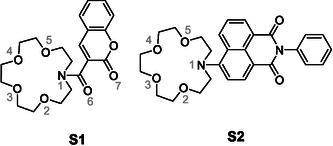
Sensors studied in this work. Numbering system for co‐ordinating atoms shown in gray.

In this study, we aim to gain insights into the structural background of crown ether‐based fluorescent sensors for Ca^2+^. We chose two benchmark sensors that are reported to show significant fluorescence enhancements on the addition of Ca^2+^. One sensor uses a PET mechanism (S1) [15] and one operates using ICT (S2) [16]; their chemical structures are shown in Figure 1. We show that both sensors undergo changes in the forms of the absorption and emission spectra on cation complexation and that these changes often vary with metal cation. We also use vibrational ion spectroscopy to show that the same sensor can form structurally distinct complexes with different metal cations—this is likely the source of the changes in spectral form. To this end, our experiments used an ion trap to isolate metal‐sensor complexes; we then recorded their vibrational spectra and compared the results with the predicted spectra from density functional theory (DFT) calculations of energy minimied structures. Finally, we investigate the effect of added water on the fluorescent response of S1 and S2 to cations and rationalize our findings using the computational prediction of partial hydration of sensor‐bound cation.

## 2 Results and Discussion

### 2.1 Fluorescence

**FIGURE 2:**
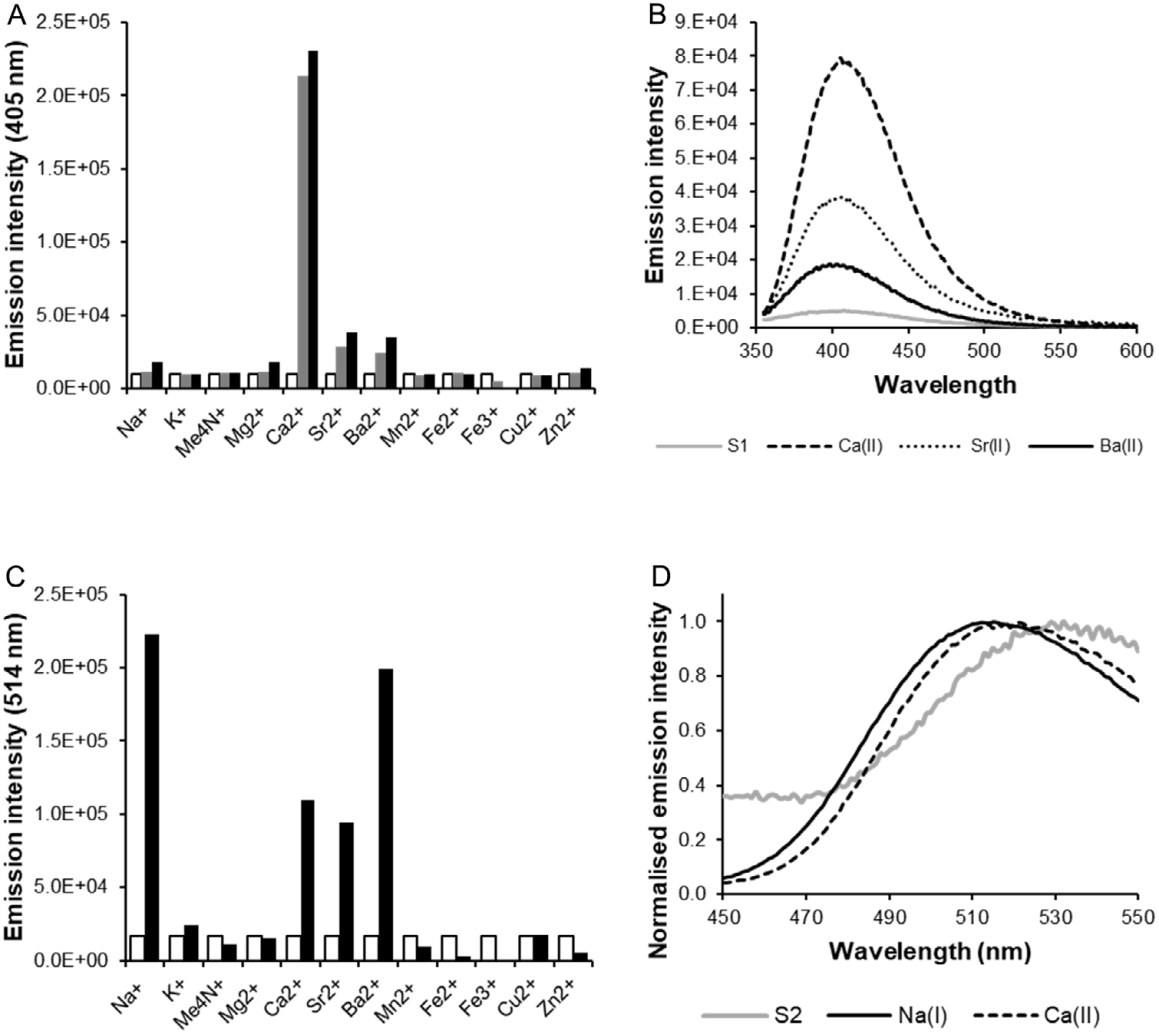
The effect of cations on fluorescence and absorption. (A) S1 (white bars) treated with 10 equiv (gray bars) or 80 equiv (black bars) of the indicated cations as perchlorate salts; [S1] = 5 μM, λ_ex_ = 315 nm. (B) Form of the emission spectra on adding saturating concentrations of cations as perchlorate salts; [S1] = 5 μM, λ_ex_ = 315 nm. (C) Form of the absorbance spectra on adding cations; [S1] = 50 μM, 20 equiv. cations added as perchlorate salts D S2 (white bars) treated with 1000 equiv (black bars) of the indicated cations as perchlorate salts; [S2] = 5 μM, λ_ex_ = 355 nm; emission with Cu^2+^ measured at 451 nm E Form of the emission spectra on adding saturating concentrations of cations as perchlorate salts; [S1] = 5 μM, λ_ex_ = 315 nm F Form of the absorbance spectra on adding cations; [S1] = 50 μM, 20 equiv. cations added as perchlorate salts. All experiments conducted in MeCN.

With S1, we confirmed the fluorescence enhancement of nearly 20x on addition of 10 equiv. Ca^2+^ (Figure 2A). We also discovered, absent from the original report, that the same level of both Sr^2+^ and Ba^2+^ gave appreciable, though much less significant, increases in emission, while Na^+^ gave a smaller enhancement still. Dipositive transition metal ions gave essentially no fluorescence enhancement, presumably because their lower ionic radii means that they are a poor fit for the crown ether. Fe^3+^ quenched fluorescence likely through a paramagnetic mechanism while the organic cation Me_4_N^+^ gave no response. None of the metals yielding a fluorescence enhancement changed the form of the emission spectrum (Figure 2B) as would be expected with a sensor based on the PET mechanism. However, the form of the absorbance spectrum was changed by both Ca^2+^ and Ba^2+^, though in subtly different ways (Figure S1A). This may imply that these metal ions form complexes with S1 that are slightly different in structure.

In our hands, the ICT sensor **S2** responded to Ca^2+^ as reported with 1000 equiv. giving an emission enhancement of ca. 7x; we also discovered that significant fluorescent responses to Sr^2+^, Ba^2+^, and Na^+^ were produced by the same amount of metal cation (Figure 2C). The response to monopositive Na^+^ is unsurprising given that fluorescent sensors for that ion based on N‐aryl aza‐15‐crown‐5 are known [17, 18]. Fluorescence intensity was not increased by any transition metal ions tested, though Cu^2+^ did shift the maximum emission wavelength (λ_em_) to 451 nm (data not shown). Uncomplexed **S2** has λ_em_ of 531 nm which shifts to 514 nm with Na^+^ but to 518 nm with group II ions (Figures 2D and S2). While this difference is subtle, the dissimilarities between the absorption spectra are quite dramatic (Figure S1) as also found in the original report [16]. This may imply a slightly different co‐ordination mode of Na^+^ compared with the group II ions and thus a different conjugation of the crown nitrogen with the fluorophore. We will return to this issue in Section 3.4.

### 2.2 Estimation of Binding Constants

Fluorescence titration experiments with S1 (Figure S3) showed that the maximal fluorescence enhancements achievable with Na^+^ and Ba^2+^ are approximately equal while less than that with Sr^2+^ which was in turn less than that with Ca^2+^. From these experiments, we used nonlinear regression (at http://app.supramolecular.org) [19] to derive logKa values (Table 1). We used a 1:1 stoichiometry for all ions as indicated by both crystal structure and Job's plot for S1 and Ca^2+^ in the original publication [15]; this yielded satisfactory fitting in all cases. The apparent binding constants reflected the fluorescence enhancements, though only in a semiquantitative manner. Thus, 80 equivalents of Na^+^ gave an enhancement of 1.8x, while the same level of Ba^2+^ gave an enhancement of only 3.5x (Figure 2) despite having a logK_a_ more than an order of magnitude greater. We also satisfactorily fitted titration data (Figure S4) for S2 to a 1:1 binding stoichiometry, but the resulting logK_a_ values (Table 1) did not show a clear correlation with fluorescence enhancements.

**TABLE 1:** Binding constants (logK_a_) of **S1** and **S2** with selected metal ions in MeCN.

	S1	S2
**Na** ^**+**^	2.23 ± 0.04	2.77 ± 0.01
**Ca** ^**2+**^	3.91 ± 0.06	2.38 ± 0.03^a^
**Sr** ^**2+**^	3.48 ± 0.13	—
**Ba** ^**2+**^	3.61 ± 0.02	2.26 ± 0.03

*Note:* Data are mean ± sd of at least two independent experiments. For representative data, see Figures S3 and S4.

a Data from [16].

**FIGURE 3:**
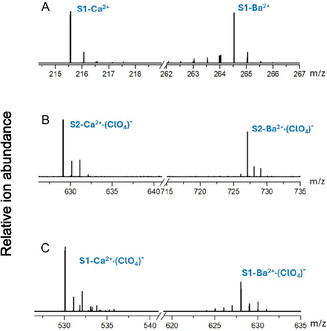
Relative sensor‐ion complex abundances by MS. Ion signals obtained in competition experiment with S1 or S2 and 5 equiv Ca^2+^ and Ba^2+^ as perchlorate salts. (A) [S1‐M]^2+^ ions. (B) [S2‐M‐ClO_4_]^+^ ions. (C) [S1‐M‐ClO_4_]^+^ ions.

In competition experiments with S1 and equimolar amounts of Ca^2+^ and Ba^2+^, we were able to detect complexes with both metal ions in approximately equal intensity (Figure 3A) indicating similar binding affinities consistent with our fluorescence titration experiments. In similar experiments with S2, we were only able to detect sensor‐ion complexes with adventitious perchlorate ions which were present with approximately equal abundance for Ca^2+^ and Ba^2+^ (Figure 3B). Assuming incorporation of perchlorate is about equally facile for both complexes this suggests very similar binding affinities, again consistent with our fluorescence titration experiments.

### 2.3 Infrared Ion Spectroscopy

**FIGURE 4:**
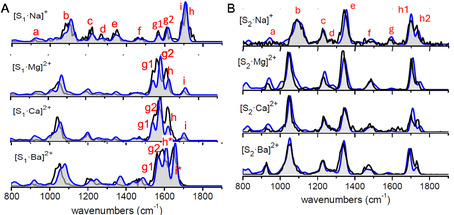
Vibrational spectra of sensor‐ion complexes. (A) S1. (B) S2. Black lines are derived from IRMPD measurements; filled blue lines are computationally predicted spectra. See text and Table 2 for assignment of bands and discussion.

The IRMPD spectra recorded for selected cation complexes of the S1 and S2 sensor molecules are depicted in Figure 4. As anticipated above, the vibrational fingerprint region 800–1900 cm^−1^ covered by the measurements, probes some of the fundamental vibrational modes of the crown ether ring and the fluorophore side chain. Indeed, the cation complexes display a characteristic progression of IRMPD spectral bands. A qualitative assignment of the most salient band structures to vibrational modes of the complexes is provided in Table 2. The bands are labeled with lower case letters a–i for discussion.

**TABLE 2:** Qualitative description of the dominant atomic motions involved in the vibrational modes associated with the IR bands observed in the spectra of the metal cation complexes of **S1** and **S2**.

	**S1‐M** ^**+**^ **/M** ^**2+**^		**S2‐M** ^**+**^ **/M** ^**2+**^
**a**	C—C stretching and C—O—C scissoring (crown ether)	**a**	C–C stretching and C—O—C scissoring (crown ether)
**b**	C—O/C—N stretching (crown ether)	**b**	C—O/C—N stretching (crown ether)
**c**	CH wagging (side group) CH_2_ twisting (crown ether)	**c**	C—N stretching (linker bond) asymm. C—N stretching (side group)
**d**	CH_2_ twisting (crown ether)	**d**	CH_2_ twisting (crown ether)
**e**	aromatic C=C stretching CH_2_ twisting C—N stretching (crown ether)	**e**	aromatic C=C stretching CH/CH_2_ wagging symm. C—N stretching (side group)
**f**	aromatic C=C stretching CH wagging (side group) CH_2_ scissoring (crown ether)	**f**	aromatic C=C stretching CH wagging (side chain) CH_2_ scissoring (crown ether)
**g1, g2**	aromatic C=C stretching CH wagging (side chain)	**g**	aromatic C=C stretching CH wagging (side chain)
**h**	C=O stretching (coumarin)	**h1**	C=O asymm. stretching CNC rocking
**i**	C=O stretching (linker)	**h2**	C=O symm. stretching CNC scissoring
h*, i* (S1‐Ba^2+^)	C=O stretching (both carbonyls) aromatic C=C stretching		

*Note:* See bands and labels in Figure 4.

The low‐frequency flank of the IRMPD spectra, roughly 800–1150 cm^−1^, is mostly associated to vibrational modes of the crown‐ether ring moiety, namely to the stretching vibrations of the C—C bonds (weak band a) and the C—O—C and C—N—C bonds (stronger band b). Band b is red‐shifted in the alkaline earth complexes with respect to the Na^+^ complex, by ca. 50 cm^−1^, due to the stronger perturbation of the covalent bonds in the crown ether backbone. The central part of the IRMPD spectrum (1150–1500 cm^−1^, bands c–f) probes bending vibrations of the methylene groups in the crown ether, along with stretching of the C—N bonds and of the aromatic C=C/C—C bonds of the fluorophore moieties. The strong band e (at ca. 1350 cm^−1^) in the S2 complexes is related to the large transition moment of the C—N stretching vibrations in the fluorophore aided by the dipole moments of the neighboring C=O groups. The high frequency region of the recorded spectra (1500–1800 cm^−1^) probes the stretching vibrations of the C=O carbonyl groups. For S2, it would be expected that the carbonyl groups modulate fluorescence but are passive structural backbone groups in terms of metal coordination. Hence, the dominant bands h1 and h2 in the spectrum, related to asymmetric and symmetric C=O stretching modes, are similar in all cation complexes. In contrast, in S1, the IRMPD spectra display in this region a comparatively richer band structure with pronounced changes across the cation series (bands g, g1, g2, h, i, h*, i*). This may imply that the C=O groups participate actively in the coordination with the metal cation. Indeed, a detailed rationalization of these bands is provided below, based on conformations and coordination arrangements, including C=O···M^n+^ coordination, predicted by the computations for the S1 cation complexes.

### 2.4 Low Energy Configurations of the Cation Complexes

**FIGURE 5:**
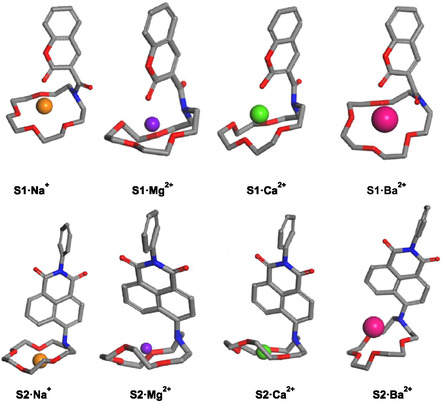
Structures of sensor‐ion complexes. Energy minimized structures which are calculated to produce vibrational spectra in accordance with experiment.

Figure 5 depicts the configurations of lowest energy predicted by the B3LYP‐D3(BJ)/def2‐TZVPPD computations for the cation complexes of S1 and S2 under study. Table 3 provides atom‐cation distances relevant to the coordination shell of each complex. Figure 4 shows that these conformations are supported by a good agreement between the corresponding computational IR spectra and the experimental IRMPD measurements.

**TABLE 3:** Distances (in Å) between the bound cation and the O and N atoms in its coordination shell, in the DFT conformations of lowest energy of the complexes considered in this study (B3LYP‐D3(BJ)/def2‐TZVPPD computations).

X‐M	**S1‐Na** ^**+**^	**S1‐Mg** ^**2+**^	**S1‐Ca** ^**2+**^	**S1‐Ba** ^**2+**^	**S2‐Na** ^**+**^	**S2‐Mg** ^**2+**^	**S2‐Ca** ^**2+**^	**S2‐Ba** ^**2+**^
**1** **N···M**	2.78 (+0.32)	2.43 (+0.25)	2.66 (+0.20)	3.67 (+0.86)	2.68 (+0.22)	2.24 (+0.06)	2.51 (+0.05)	2.88 (+0.07)
**2** **O···M**	2.30 (−0.05)	2.10 (+0.03)	2.33 (−0.02)	2.79 (+0.09)	2.32 (−0.03)	2.09 (+0.02)	2.31 (−0.04)	2.64 (−0.06)
**3** **O···M**	2.35 (0.00)	2.11 (+0.04)	2.41 (+0.06)	2.79 (+0.09)	2.42 (+0.07)	2.12 (+0.05)	2.39 (+0.04)	2.74 (+0.04)
**4** **O···M**	2.37 (+0.02)	2.15 (+0.08)	2.39 (+0.04)	2.68 (−0.02)	2.38 (+0.03)	2.10 (+0.03)	2.35 (0.00)	2.68 (−0.02)
**5** **O···M**	2.33 (−0.02)	2.10 (+0.03)	2.35 (0.00)	2.97 (+0.27)	2.34 (−0.01)	2.11 (+0.04)	2.40 (+0.05)	2.75 (+0.05)
**6** **C=O···M**	2.24 (+0.11)	1.98 (−0.09)	2.24 (+0.11)	2.61 (−0.09)				
**7** **C=O···M**	4.10 (+1.65)	4.00 (+1.93)	4.27 (+1.82)	2.72 (+0.02)				

*Notes:* See Figures 1 and 5 for a graphical representation of the molecular structures and the labels 1–7 employed to denote the cation‐atom distances. The values in brackets indicate the difference between each distance and the sum of the radii of the interacting cation and atom (Shannon–Prewitt effective radii are considered for reference: 1.00, 0.72, 1.00, 1.35, 1.35, and 1.46 Å, for Na^+^, Mg^2+^, Ca^2+^, Ba^2+^, O^2−^ and N^3−^, respectively [20, 21].).

In S2, cation binding predominantly relies on coordination with the polar O‐ and N‐ atom centers in the crown ether ring. Table 3 reveals that the coordination distances are close to the values expected for the sum of the effective electronic radii of the cation and atoms involved. For the alkaline‐earth cations, the aromatic moiety adopts a rotated configuration that also contributes to binding through cation‐π interactions [22]. This type of interaction has been previously observed in related polyaromatic systems, e.g., for naphthalimide and Hg^2+^ [23]. The beneficial role of the cation‐π interaction in the S2 complexes becomes also apparent from the computational prediction that cation binding on the face of the crown ether cavity opposite to the aromatic side group, while allowing a more relaxed side chain orientation, leads to configurations typically about 2–6 kJ/mol higher in energy and are associated with IR spectra that are at odds with experiment (see Figure S5). The only divergence is found in the S2‐Na^+^ complex, in which the cation coordinates weakly with the N‐atom of the crown (at a distance ca. 0.2 Å larger than expected from electrostatics) and with the π cloud of the fluorophore. These weaker interactions of the Na+ cation likely explain why the UV‐Vis absorption spectrum of the S2‐Na^+^ complex is dramatically different from that of the alkaline‐earth complexes (Figure S1).

The scenario is qualitatively different in S1, where the C=O groups of the linker and of the coumarin moiety lie in the vicinity of the cation docking site and play a relevant role in the coordination process. Figure 5 and Table 3 show that, in all complexes, the coumarin C=O group is oriented towards the cation and at a coordination distance within 0.1 Å of the expectation form the corresponding electronic radii. This is consistent with the involvement of the coumarin carbonyl in binding the metal ion in the solid state of the S1‐ Ca^2+^ complex [15] and earlier work on a very similar compound [24]. In contrast, the approach of the linker C=O group to the cation is restricted, due to repulsive carbonyl‐carbonyl interactions. This second C=O group only enters the coordination shell in the S1‐ Ba^2+^ complex, as the cation becomes large enough to shield the carbonyls from each other. Table 3 shows that the carbonyl‐cation interactions are optimized for all alkaline‐earth cations with a corresponding lengthening of the distance of the cation to the N‐atom of the crown ether ring. For the Ba^2+^ complex, the coordination with the two C=O groups also reduces the interaction with one of the oxygen atoms of the ring (O5) which now lies at a longer distance (+0.27 Å).

As anticipated above, the participation of the C=O side groups is also apparent in the recorded IR spectra. The high‐frequency flank of the IR spectrum (1500–1800 cm^−1^, Figure 4) provides a sensitive probe for carbonyl‐cation interactions. The presence of a distinct high frequency band at 1725 cm^−1^ (band i) is indicative of a free (non‐interacting) C=O group in the S1‐ Mg^2+^ and S1‐ Ca^2+^ complexes. This band is substituted in the S1‐ Ba^2+^ complex by two red‐shifted components at 1610 and 1670 cm^−1^ (bands h*, i*) due to the concerted stretching of the two carbonyl groups now both coordinating to the Ba^2+^ cation. This difference in co‐ordination modes plausibly explains the differences we observe between the UV absorption spectra of S1‐Ca^2+^ and ‐Ba^2+^ complexes (Figure 2C). It also potentially explains why our MS competition experiments show ca. twice the abundance of S1‐ Ca^2+^ with adventitious perchlorate as the corresponding—Ba^2+^ complex (Figure 3C)—the additional carbonyl co‐ordination to the metal ion impedes perchlorate association.

### 2.5 Effect of Water on Fluorescent Response

**FIGURE 6:**
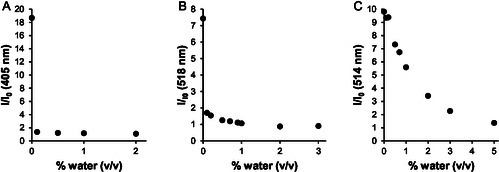
Addition of water reduces fluorescent response of sensors to metal ions. (A) [S1] = 5 μM, [Ca^2+^] = 100 μM in MeCN, λ_ex_ = 315 nm. (B) [S2] = 5 μM, [Ca^2+^] = 5 mM in MeCN, λ_ex_ = 355 nm. (C) [S2] = 5 μM, [Na^+^] = 5 mM in MeCN, λ_ex_ = 355 nm.

Finally, we turned our attention to the water sensitivity of the sensors and discovered that, for both S1 and S2, addition of only 0.1% v/v water was sufficient to destroy the fluorescent response to Ca^2+^ in acetonitrile (Figure 6A,B) despite fluorescence from the sensors alone showing very little dependence on water content at similar levels (Figure S6). Similar observations have previously been reported for an ICT calcium sensor based on an N‐aryl aza‐crown [25]. The large dehydration energy of Ca^2+^ (1550 kJ mol^−1^, ref. [26]) may be a factor as, unless this is matched by a similarly high energy of complexation, then Ca^2+^ will tend to remain hydrated in solution, not bind to sensor and so produce no fluorescent response. However, this is unlikely to be a complete explanation as the fluorescence from S2 is reduced marginally less than that from S1 (Figure 6) despite having a significantly lower Ca^2+^ binding constant (Table 1). An alternative explanation would be that H_3_O^+^ present in the water competes with Ca^2+^ for binding to the crown ether. We consider this unlikely as neither absorbance nor fluorescence vary with pH in the range 3–10 for either sensor (Figure S7), and the mass spectrometry experiments showed no evidence of the stabilization of H_3_O+ complexes upon electrospray ionization.

**FIGURE 7:**
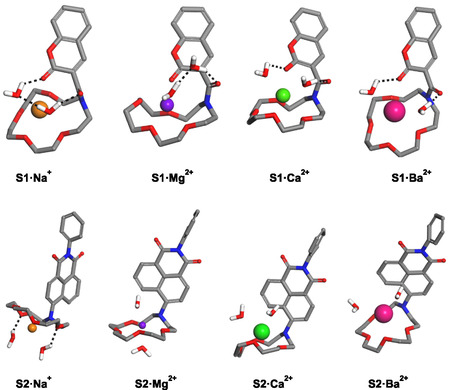
Structures of hydrated sensor‐ion complexes. Calculation of energy minimized structures shows that the metal ion in each complex can be hydrated with minimal perturbation of sensor co‐ordination but significantly different hydration geometries.

Computation provided a more plausible explanation. Figure 7 depicts the configurations of lowest energy found in our conformational survey for cation complexes microsolvated by two explicit water molecules. We found that the alkaline‐earth complexes of both S1 and S2 could undergo a facile hydration of the metal ion positioning water molecules in close proximity to the fluorophore. This is likely to activate a nonradiative decay pathway whereby electronic excitation is transferred to vibrations of O—H bonds [27] with the consequence that fluorescence is reduced. The reason low percentages of water had only negligible effects on fluorescence from sensor alone (Figure S6) was that under those conditions, water was free in solution and so much less likely to encounter a sensor molecule. Complexation of metal cation by the sensor creates a template for water‐fluorophore interactions. (This is reminiscent of lanthanide complexes where emission is reduced by a bound water molecule and correspondingly enhanced by the displacement of that water by anions [28, 29].) We tested our understanding by examining the water sensitivity of the response of S2 to Na^+^. Given that water molecules are predicted to bind the S2‐Na^+^ complex without perturbing ion binding but on the opposite side of the crown ether from the fluorophore (Figure 7), we expected that the fluorescence response would show a much less significant dependence on water content. Experiment confirmed our expectation (Figure 6C)—addition of water generating a much less dramatic reduction in fluorescence response to Na^+^ supporting the idea that partial hydration of ion bound to the sensor contributes to the reduced response to Ca^2+^.

## 3 Conclusion

We have investigated the performance of benchmark Ca^2+^ fluorescent sensors reported in previous studies to operate through PET (S1) and ICT (S2) mechanisms. Their optical absorbance and emission responses in interaction with alkali, alkaline‐earth and transition metal cations have been assessed and their sensitivity to their exposure to water has been evaluated. Finally, we have combined IRMPD spectroscopy and DFT modeling of the isolated ion complexes to rationalize the observed sensor performances from first principles. It has been shown that these techniques provide access to coordination structures of sensor‐ion complexes; to our knowledge this is the first time that such structural information has been provided for these systems in the solution phase. The extreme sensitivity of mass spectrometry allows us to isolate and characterize even sensor‐ion complexes with low binding constants. This supports the apparent disconnect between fluorescence enhancement and binding energy; further work is needed to understand why these two parameters do not appear to be related in a simple way. Until this puzzle is solved the rational design of fluorescent sensors for metal cations, especially those that are selective for a given ion, will remain challenging.

Nevertheless, our work does suggest some pointers for improved sensor design. It may be possible to enhance selectivity for divalent cations by positioning the fluorophore close to the ion binding site thus designing in cation‐π interactions between the fluorophore and the ion, as in Figure 5. Conversely sensors that retain their sensitivity in water may result from designs that separate the ion from the fluorophore thus preventing ion‐assisted association of water molecules that quench fluorescence. It is perhaps noteworthy in this respect that the only polyether‐based Ca^2+^ sensors reported to be tolerant of double digit water content have their fluorophores and ionophores spatially separated [30, 31].

## 4 Experimental Section

### 4.1 Materials

All chemicals were obtained from commercial suppliers and used without purification unless otherwise stated. Acetonitrile (MeCN) for spectroscopy was stored over activated 4 Å molecular sieves. Small portions of metal perchlorate salts were freeze dried and stored in a desiccator. 1 M stock solutions in acetonitrile were stored at 4°C and dilutions prepared as required. **S1** and **S2** were stored as 1 mM solutions in MeCN at 4°C and diluted as necessary.

### 4.2 Synthesis

We synthesized samples of each sensor using the previously published routes [15, 16].

### 4.3 Absorbance and Fluorescence Spectroscopy

Photophysical experiments were conducted in a quartz cuvette using an Edinburgh Instruments FS5 fluorimeter with Fluoracle software. Step size was 1 nm and dwell time was 0.2 s; excitation and emission slit widths were 2 nm. Excitation and emission wavelengths are given in the main text.

The effect of water on cation‐induced emission from sensors was assessed by the sequential addition of aliquots of water to solutions containing the state concentrations of sensor and metal perchlorate salt; total added volume did not exceed 5% of the volume of sensor and cation perchlorate solution.

Binding titration experiments were performed by the sequential addition of aliquots of solutions of metal perchlorate salts to 5 μM solutions of sensor; total added volume did not exceed 5% of the volume of sensor solution. Binding constants were calculated using nonlinear regression with BindFit (http://app.supramolecular.org/bindfit/) [19], and the fits have been made publicly available (see the Supporting Information for details).

The effect of pH on sensor absorbance and emission was assessed in a 1:1 mixture of acetonitrile and 10 mM HEPES adjusted to the indicated pH.

### 4.4 Mass Spectrometry Competition Experiments

Electrospray ionization mass spectrometry was employed to estimate binding affinities of cations and sensor molecules from the detected relative abundances of the corresponding complexes. The ability of electrospray ionization to transfer noncovalent complexes from solution to the gas‐phase has been reviewed in different works [32, 33, 34]. The cations Ca^2+^ and Ba^2+^ were employed as benchmarks for these measurements. In order to promote competitive binding, each fluorophore sensor, **S1** or **S2** (∼10 μM), was combined with comparably higher concentrations of perchlorate salts of the two cations (∼50 μM), with acetonitrile as solvent. The analysis was performed in an orbitrap mass spectrometer (Q‐Exactive Focus, Thermo Scientific). The solution was injected at a flow rate of 5 mL/min and the electrospray emitter was operated at 3 kV voltage and 270°C capillary temperature.

### 4.5 Vibrational Ion Spectroscopy

Vibrational spectroscopy of the gaseous ion complexes was performed with the Infrared multiple‐photon dissociation (IRMPD) technique at the free‐electron laser facility FELIX (Nijmegen, The Netherlands) [35]. IRMPD spectra were recorded for complexes of **S1** and **S2** with Na^+^, Mg^2+^, Ca^2+^, and Ba^2+^, isolated at room temperature in an ion trap (Bruker AmaZon Speed). The complexes were produced by electrospray ionization of acetonitrile solutions of one sensor compound **S1** or **S2** (∼100 μM) and the corresponding cation chloride salt (∼50 μM). The resulting cationic complexes, singly charged for Na^+^ and doubly charged for the alkaline‐earth cations, were mass‐isolated in the quadrupole ion trap mass spectrometer for spectroscopic interrogation [36].

The IRMPD experiments covered the spectral range 800–1900 cm^−1^, in order to probe vibrational modes of the crown ether moiety and the carbonyl and aromatic side groups (e.g., C—C/C=C, C—N, C—O, C=O stretching and CH_2_ bending vibrational modes). The ions were irradiated with infrared free‐electron laser pulses of 60–90 mJ energy. The spectral laser bandwidth amounts to ∼0.5% of the central IR frequency. The laser frequency is scanned at discrete steps of 5 cm^−1^, and when it matches a vibrational transition of the ion complex, multiple photon absorption occurs, resulting in resonant fragmentation. One main product ion of the IRMPD process, common for both sensor complexes, involves the cleavage of the side fluorophore, leaving the aza‐crown moiety bound to the metal cation. Further common product ions are related to a partial fragmentation of the side chain. The IRMPD spectrum was built from the depletion of precursor complex ion (I_P_), and the integrated product ion intensities (I_F_), by plotting −ln(I_P_/[I_P_+I_F_]) as a function of the IR frequency. Linear normalization was applied to account for changes in the laser energy during scans. Further details of the IRMPD methodology and the FELIX facility can be found in previous publications [36, 37].

### 4.6 Quantum Chemical Modeling

The low‐energy conformations of the cation complexes of the molecular sensor probes were modeled within the framework of density functional theory (DFT). The functional B3LYP‐D3(BJ) [38] was employed with the basis set def2‐TZVPPD [39, 40]. Candidate structures were generated from a sampling of the conformational landscapes of the cation complexes with the Conformer–Rotamer Ensemble Sampling Tool (CREST) [41]. The configurations were ranked according to the zero‐point corrected electronic energies predicted by the DFT computation. For comparison with the spectroscopy experiments, the computational IR spectra were generated by convoluting the normal modes of the conformers with a Gaussian line broadening of 25 cm^−1^ (fwhm), with the DFT vibrational frequencies scaled by a factor of 0.97 to effectively correct for anharmonic spectral shifts [42].

The same computational methodology outlined above was applied to cation complexes hydrated by two explicit water molecules. According to our conformational search, this was the maximum number of water molecules that could interact directly with the bound cations through H_2_O···M bonds in low energy configurations; the incorporation of a third water molecule either contributed to the second hydration shell of the bound cation or led to hydration of the C=O carbonyl groups of the side chain and intervened only indirectly in cation binding.

## Funding

This work was supported by the Royal Society of Chemistry (U24‐3976923672) and the HORIZON EUROPE Innovative Europe.

## Conflicts of Interest

The authors declare no conflicts of interest.

## Supporting information

Supplementary Material

## Data Availability

The data that support the findings of this study are available from the corresponding author upon reasonable request.
