# Supplementary material for: Rational Design of Crown Ether‐Based Fluorescent Sensors for Group II Cations: Insights From Vibrational Spectroscopy and Computation
Source: Chemphyschem. 2026 Jun 29;27(12):e202500770. doi: 10.1002/cphc.202500770 (PMC13314387; doi:10.1002/cphc.202500770)
Supplement: Supplementary file 1 — Supplementary Material [file CPHC-27-e202500770-s001.pdf]

Supporting information for:

Rational design of crown ether-based fluorescent sensors for group II cations: insights from vibrational spectroscopy and computation

Bruno Martinez-Haya (0000-0003-2682-3286),*[a] Jennifer C. Anene,[b] Laura Finazzi (0009-0009-5675-7890),[c] Jos Oomens (0000-0002-2717-1278),[c] Simon Wheeler (0000-0003-0215-8648)*[b]

[a] Department of Physical, Chemical and Natural Systems, Universidad Pablo de Olavide, 41013, Seville, Spain; bmarhay@upo.es
[b] Leicester School of Pharmacy, De Montfort University, The Gateway, Leicester, LE1 9BH, UK; simon.wheeler@dmu.ac.uk
[c] Institute for Molecules and Materials, FELIX Laboratory, Radboud University, 6525ED, Nijmegen, The Netherlands

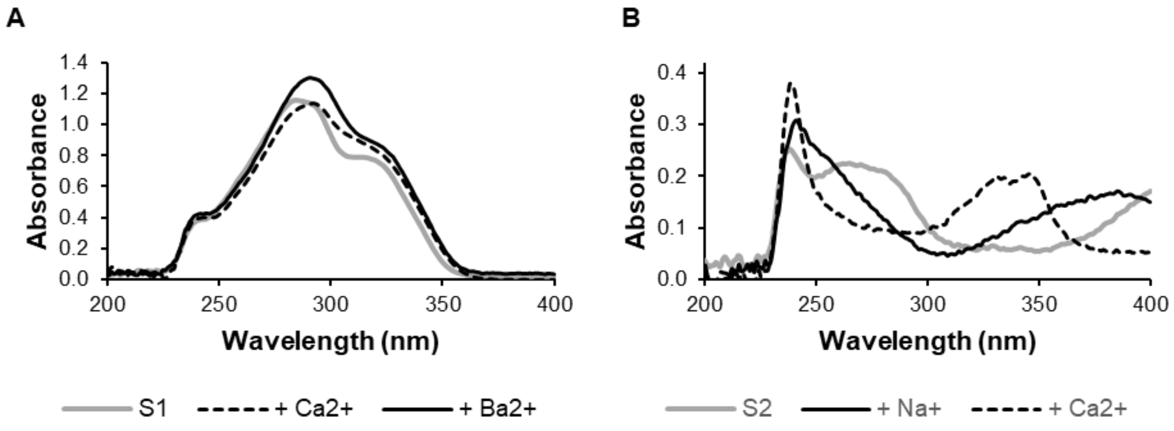

Figure S1 The effect of cations on absorption A Form of the absorbance spectra on adding cations; [S1] = 50 μM, 20 equiv. cations added as perchlorate salts **B** Form of the absorbance spectra on adding cations; [S2] = 50 μM, 1000 equiv. cations added as perchlorate salts. All experiments conducted in MeCN.

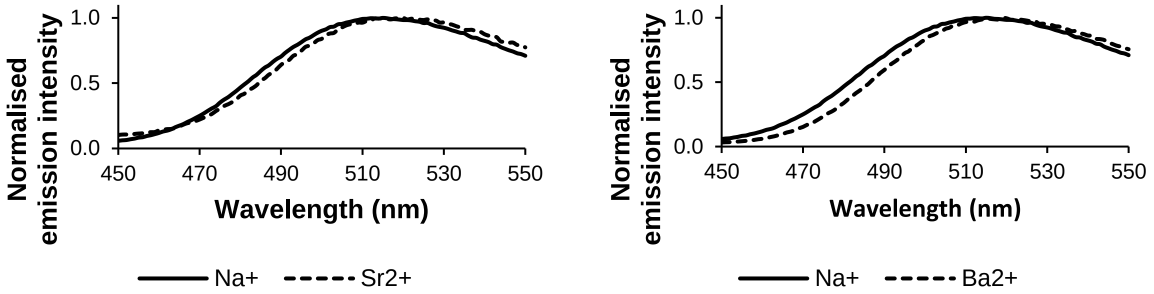

Figure S2 S2 gives subtly different λ_{em} with Na^+ than with group II ions [S2] = 5 μM in MeCN; cations added as perchlorate salts, λ_{ex} = 355 nm.

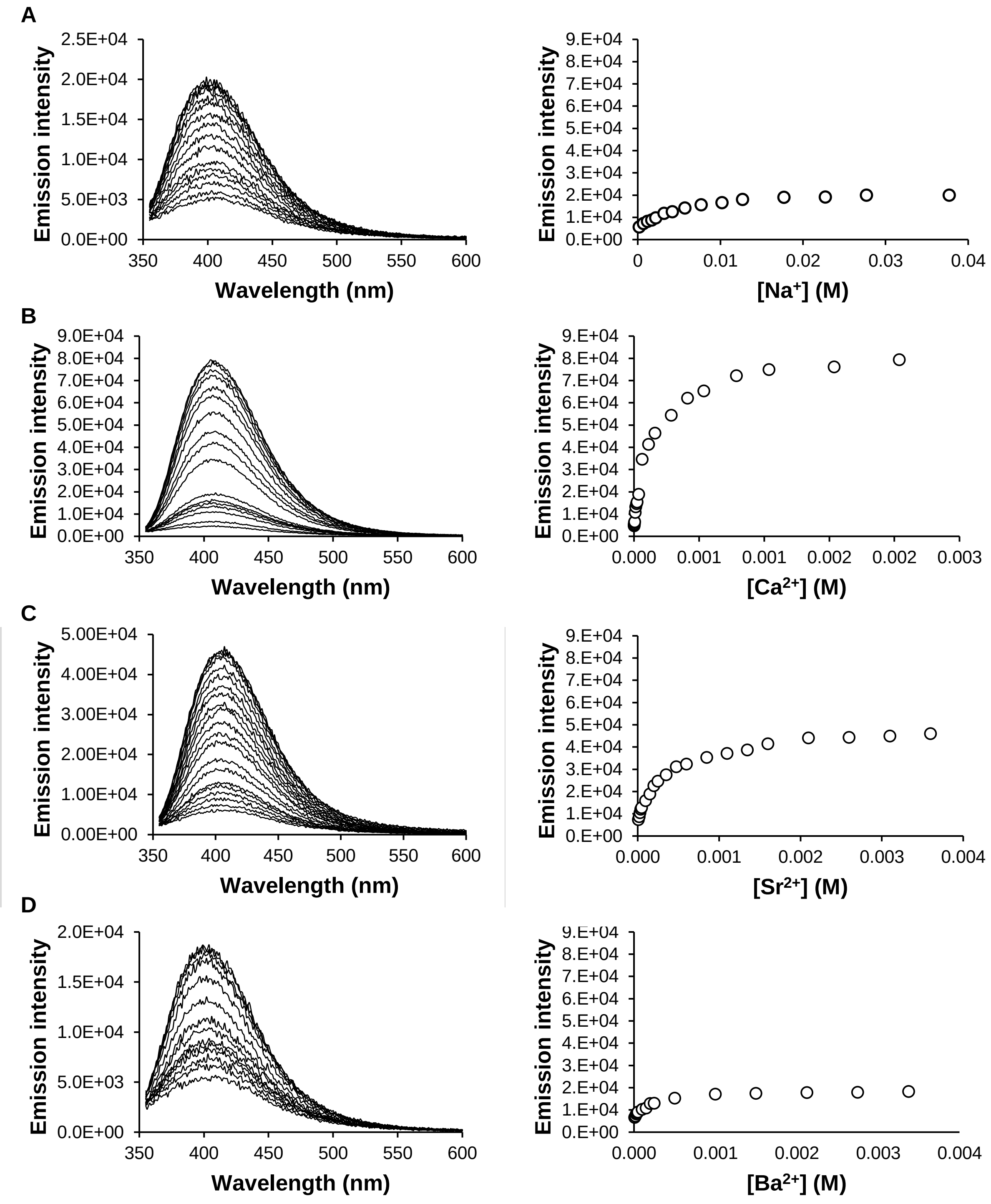

Figure S3 Binding titration experiments between S1 and selected cations A Na^{+} **B** Ca^{2+} **C** Sr^{2+} **D** Ba^{2+} Data shown are representative examples of at least two independent experiments. All experiments were conducted in acetonitrile; [S1] = 5 μM; λ_{ex} = 315 nm. Titration plots show emission at 405 nm.

Representative data and fits are publically available: Na+, Ca^{2+}, Sr^{2+}, Ba^{2+}

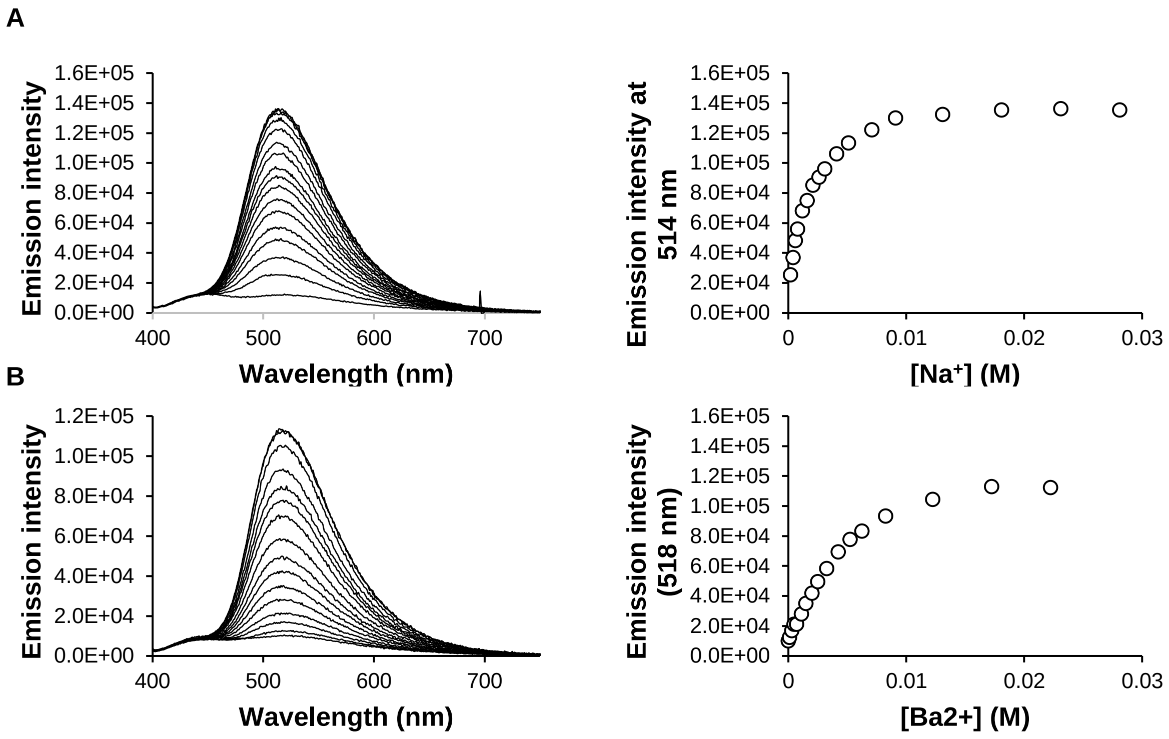

Figure S4 Binding titration experiments between S2 and selected cations A Na^+ **B** Ba^{2+} Data shown are representative examples two independent experiments. All experiments were conducted in acetonitrile; [S2] = 5 μM; λ_{ex} = 355 nm.

Representative data and fits are publically available: Na+, Ba^{2+}

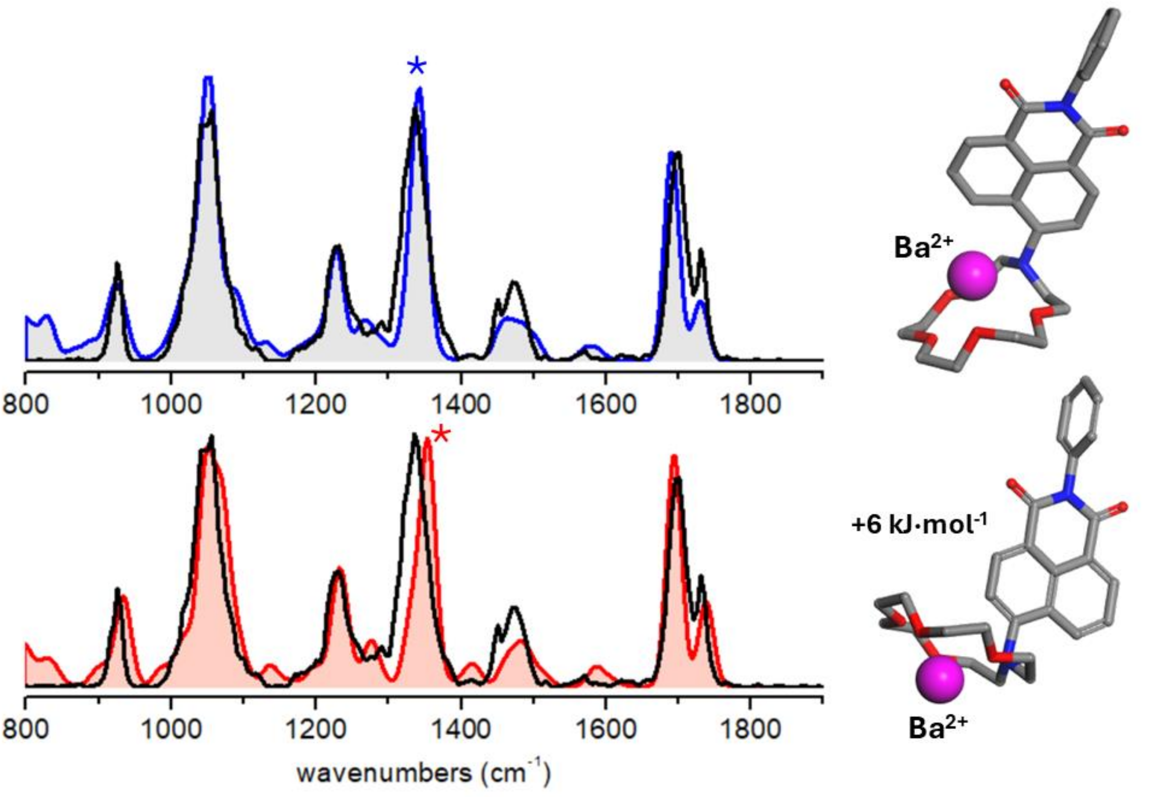

Figure S5: Binding of Ba^{2+} next to the fluorophore is aided by cation-π interactions. Binding on the opposite side of the crown ether cavity is less stable (by 6 kJ·mol^{-1} in the present computations) and leads to a sizeable shift of the vibrational band at ~1350 cm^{-1} that is at odds with the recorded IR spectrum. Therefore, this latter configuration can be ruled out in the present experiments. See Table 2 of the paper for an assignment of the vibrational bands). The experimental spectrum is drawn as a black trace, while the computational spectra are represented by a coloured shaded trace.

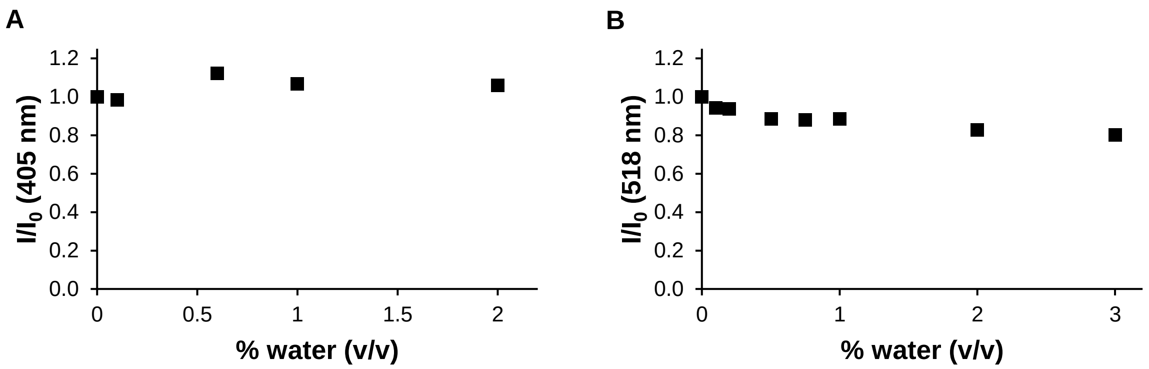

Figure S6 Addition of water does not significantly affect fluorescence of sensors **A** [S1] = 5 μM in MeCN, λ_{ex} = 315 nm **B** [S2] = 5 μM in MeCN, λ_{ex} = 355 nm.

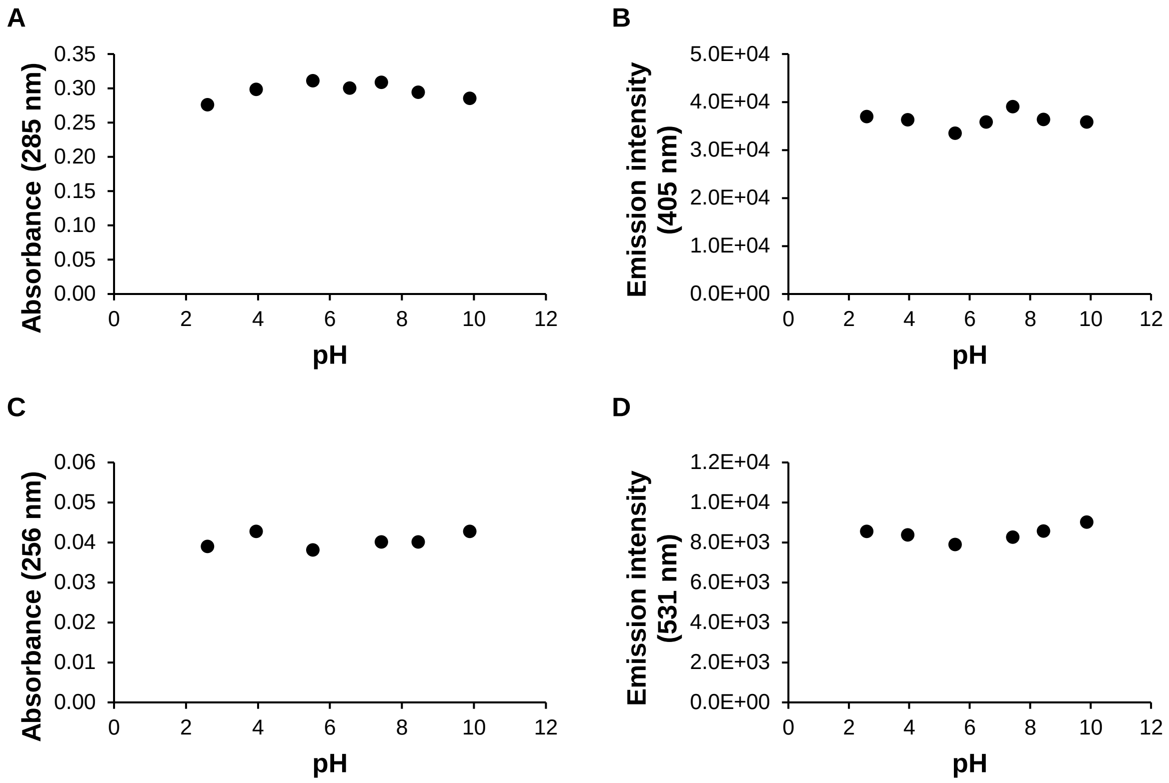

Figure S7 Absorbance and emission variations with pH **A** S1 absorbance **B** S1 emission **C** S2 absorbance **D** S2 emission. Experiments conducted in 1:1 MeCN/10 mM HEPES adjusted to pH indicated, [S1], [S2] = 5 μM. For fluorescence experiments λ_{ex} = 315 nm for **S1**, λ_{ex} = 355 nm for **S2**.
